# Demographic-based disparities in outcomes for adults with central line-associated bloodstream infections in the United States: a National Inpatient Sample database study (2016–2020)

**DOI:** 10.3389/fmed.2024.1469522

**Published:** 2024-10-11

**Authors:** Marie Dix, Troy Belleville, Anjali Mishra, Ryan W. Walters, Paul Millner, Ali Bin Abdul Jabbar, Abubakar Tauseef

**Affiliations:** ^1^Creighton University School of Medicine, Omaha, NE, United States; ^2^Creighton University Department of Clinical Research and Public Health, Omaha, NE, United States; ^3^Creighton University Department of Internal Medicine, Omaha, NE, United States

**Keywords:** CLABSI, hospital-acquired infections, length of stay, in-hospital mortality, hospital cost, National Inpatient Sample, database, disparities (health racial)

## Abstract

**Introduction:**

Central line-associated bloodstream infections (CLABSI) are prevalent and preventable hospital-acquired infections associated with high morbidity and costs. Disparities based on race, ethnicity, and hospital factors remain underexplored. This study compares cost, length of stay, and mortality for adults with CLABSI by race-ethnicity, hospital location-teaching status, and geographic region in the United States using data from the National Inpatient Sample (NIS) database from 2016 to 2020.

**Methods:**

The hospitalization cohort included adults diagnosed with CLABSI, excluding those with primary CLABSI diagnoses, cancer, immunosuppressed states, or neonatal conditions. Primary outcomes were in-hospital mortality, length of stay, and hospital costs, adjusted to mid-year 2020 US dollars. Independent variables included race-ethnicity, hospital location-teaching status, and geographic region. All analyses accounted for NIS sampling design.

**Results:**

From 2016 to 2020, there were approximately 19,835 CLABSI hospitalizations. The overall in-hospital mortality rate was 9.1%, with a median hospital stay of 16.9 days and median cost of $44,810. Hispanic patients experienced significantly higher mortality, longer length of stay, and higher costs compared to non-Hispanic Black and White patients. Urban teaching hospitals had longer stays and higher costs than rural and urban non-teaching hospitals. Regionally, the Northeast and West had higher costs and longer stays than the Midwest and South, but mortality rates did not differ significantly.

**Conclusion:**

This study highlights significant disparities in CLABSI outcomes based on demographic factors. Addressing these disparities is crucial for improving CLABSI management and healthcare equity. Further research should explore the underlying causes of these differences to inform targeted interventions.

## 1 Introduction

Central line-associated bloodstream infections (CLABSI) are common and largely preventable hospital-acquired infections associated with high morbidity and cost. The joint commission defines a CLABSI as “a primary bloodstream infection that develops in a patient with a central line in place within the 48 h before onset of the bloodstream infection that is not related to infection at another site” (1). Infection can be intraluminal or extraluminal, and the causative pathogens often form biofilms around the central line which allows them to resist host defenses and antimicrobial treatments (2).

CLABSI represent a large burden to the healthcare system and a threat to patient safety. Its incidence varies among population but has been estimated to be between 0.5 and 5 per 1,000 catheter days (3). In 2022, a total of 30,828 cases of CLABSI were reported from acute care and critical access hospitals in the United States (4). Patients with a central venous catheter who develop CLABSI are 36.6% more likely to die in the hospital, 37.0% more likely to be readmitted, and have an average hospital stay increase of 2 days compared to those without CLABSI (5). The attributable cost of these infections has been estimated to be $11,971 in the adult population (6).

The magnitude of the issue has sparked quality improvement initiatives which have mitigated risk from systemic factors, including duration of catheterization, catheter type and site, and substandard catheter care (3, 7). This may explain the 9% decrease in CLABSI cases in acute care hospitals between 2021 and 2022 (3, 4). However, these interventions do not address patient-centered risk factors, such as coagulopathies, dementia, diabetes, drug abuse, hemiplegia and paraplegia, HIV/AIDS, malignancy, severe liver disease, obesity, renal disease, and malnutrition (3, 8). No existing study analyzing CLABSI in adults accommodates for these co-morbidities.

The effect of patient-centered demographic factors is an intriguing topic of study as our nation pursues health equity. There has been an increasing prevalence of CLABSI reported among Hispanic and non-Hispanic Black patients as compared to non-Hispanic White patients (9, 10), though this has not been uniformly replicable across healthcare systems (11). The interplay between race/ethnicity and length of stay, cost, and mortality remains to be explored.

The effect of hospital location on outcomes for CLABSI patients is also poorly understood. Although rural hospitals are more often challenged by limited resources than their urban counterparts, a study of 218 hospitals in California found no association between rural location and CLABSI rate (12). There has yet to be a nationwide study that investigates the impact of CLABSI on patient outcomes among geographic regions of the United States.

This study investigates the differences in cost, length of stay, and mortality rate for patients with CLABSI by racial groups, hospital location-teaching status, and hospital region of the United States using adult inpatient data from the 2016–2020 National Inpatient Sample (NIS) database.

## 2 Materials and methods

### 2.1 Data source

Hospitalization data were abstracted from the 2016 to 2020 National Inpatient Sample (NIS), which is part of a family of databases for the Healthcare Cost and Utilization Project (HCUP) developed in a partnership with the Agency for Healthcare Research and Quality (AHRQ). The NIS approximates a 20% stratified sample of all discharges from community hospitals in the United States, excluding rehabilitation and long-term acute care hospitals. The NIS covers more than 97% of the United States population and when weighted contains more than 35 million yearly discharges (13).

### 2.2 Hospitalization cohort

Because the NIS does not provide present-on-admission indicators, patient safety indicator 07 from AHRQ was used as a guide to identify hospitalizations with suspected hospital associated CLABSI (14). Specifically, we included hospitalizations with a diagnosis of CLABSI in which the patient was at least 18 years of age and a minimum of two-day length of stay. We excluded hospitalizations for which CLABSI was the primary diagnosis as well as hospitalizations in which the patient had a diagnosis of cancer or immunosuppressed state, a major diagnostic category for newborn and other neonates (MDC 15), or an ungroupable Medicare-severity diagnosis-related group (MS-DRG 999). All ICD-10 diagnosis and procedure codes for this study are provided in Supplementary Table S1.

### 2.3 Outcomes

Primary outcomes included all-cause in-hospital mortality, length of stay, and hospital cost. Hospital cost was inflation-adjusted to mid-year 2020 US dollars (15). Secondary outcomes included in-hospital complications associated with CLABSI that included sepsis, septic shock, severe sepsis, acute myocardial infarction, infective myocarditis, infective pericarditis, acute and subacute endocarditis, cardiogenic shock, septic arterial embolism, cerebral infarction, acute respiratory distress syndrome (ARDS), pneumonia, pulmonary embolism, urinary tract infection (UTI), polyneuropathy, osteomyelitis, pyogenic arthritis, acute kidney injury (AKI), mild cognitive impairment, altered mental state, bile duct obstruction, acute pancreatitis, disseminated intravascular coagulation, vein thrombosis, phlebitis and thrombophlebitis, and antibiotic resistance (Supplementary Table S1).

### 2.4 Independent variables and covariates

Primary independent variables included race-ethnicity (non-Hispanic White, non-Hispanic Black, Hispanic, and other [that included Asian or Pacific Islander, Native American, and other races]), hospital location-teaching status (rural, urban non-teaching, urban teaching), and hospital geographic region as defined by the United States Census Bureau (Northeast, Midwest, South, West). The NIS combines race and ethnicity information into a single variable; thus, a patient of Hispanic ethnicity of any race are considered Hispanic (allows use of the non-Hispanic adjective). Further, the decision to combine hospitalizations in which the patient was Asian or Pacific Islander or Native American with the other race category was due to low observed hospitalizations counts that would have violated the NIS Data Use Agreement for most of the variables of interest. In addition, hospital location status is based upon Core Based Statistical Area (CBSA) defined by the United States Office of Management and Budget with standards based on the 2010 census, whereas hospital teaching status is defined by presence of approved residency programs, being a member of the Council of Teaching Hospitals (COTH), or a ratio of full-time equivalent interns and residents to beds of 0.25 or higher.

Covariates included patient age, biological sex, primary payer (Medicare, Medicaid, private, other which includes uninsured [self-pay] and other government insurance programs [e.g., TRICARE]), weekend admission status, alcohol dependence, asplenia, burns, coronary arterial disease (CAD), history of malignancy, cerebral palsy, chronic corticosteroid use, liver cirrhosis, chronic kidney disease, congestive heart failure, Crohn’s disease, ulcerative colitis, cystic fibrosis, dementia, depression, diabetes, dyslipidemia, gallstones, gastroesophageal reflux disease, hemodialysis status, HIV or AIDS, hypertension, irritable bowel syndrome, ischemic heart disease, left ventricular systolic dysfunction, malnutrition, need for mechanical ventilation, history of myocardial infarction, nephrolithiasis, obesity, paralysis, pulmonary hypertension, peripheral vascular disease, sickle cell disease, spinal cord injury, stroke, transient ischemic attack, and history of organ transplant (Supplementary Table S1). We also quantified comorbidity burden using the Charlson Comorbidity Index (CCI) (16).

### 2.5 Statistical analysis

All descriptive statistics are stratified separately by race-ethnicity, hospital location-teaching status, and hospital geographic region. Continuous variables are presented as median and interquartile range, whereas categorical variables are presented as percent. The HCUP data use agreement precludes presenting percentages when unweighted hospitalization counts are less than 11; this is indicated in our results with an asterisk (17). Continuous variables were compared between cohorts using a linear regression model, whereas categorical variables were compared using the Rao-Scott chi-square test. Between-stratification differences for in-hospital death were evaluated using unadjusted and adjusted logistic regression models, whereas LOS and inflation-adjusted hospital cost were compared using unadjusted and adjusted lognormal regression models. Adjusted models included race-ethnicity, hospital location-teaching status, hospital geographic region as well as patient age, biological sex, primary payer, comorbidity burden, weekend admission status, coronary arterial disease, depression, dyslipidemia, hypertension, malnutrition, obesity, sepsis, septic shock, severe sepsis, acute myocardial infarction, cerebral infarction, cardiogenic shock, ARDS, disseminated intravascular coagulation, pneumonia, UTI, AKI, acute pancreatitis, vein thrombosis, acute and subacute endocarditis, and pulmonary embolism. The functional form of continuous covariates was evaluated using restricted cubic splines with knot points at the 5, 35, 65, and 95th percentiles; the decision to retain non-linear forms was dictated by likelihood ratio test (18). Further, multicollinearity of all covariates was evaluated using variance inflation factors (VIF), with a VIF greater than 4 indicating a potential collinearity issue. All analyses were conducted using SAS v. 9.4 and accounted for the NIS sampling design. NIS weighting was used when extrapolating descriptive statistics to the national level. Two-tailed *p* < 0.05 was used to indicate statistical significance.

## 3 Results

### 3.1 Overall hospitalization cohort

From 2016 through 2020, there were an estimated 19,835 hospitalizations in the United States that included CLABSI per our inclusion criteria (95% CI: 19,014 to 20,656). Within these hospitalizations, 51.8% were male, the median age was 56 years (IQR: 39 to 67, range: 18 to ≥90), 45.0% were Medicare beneficiaries, 26.5% were Medicaid beneficiaries, 21.4% had private insurance (Supplementary Table S2). Further, the most common comorbid conditions included hypertension (48.3%), diabetes (32.4%), dyslipidemia (25.3%), obesity (24.1%), history of CAD (21.4%), congestive heart failure (20.3%), and malnutrition (19.2%; Supplementary Table S2).

The overall mortality rate was an estimated 9.1% (95% CI: 8.1 to 10.0%), median hospital length of stay was an estimated 16.9 days (95% CI: 16.4 to 17.4), and median hospital cost was an estimated $44,810 (95% CI: $43,267 to $46,407). The most common complications included sepsis (61.1%), AKI (44.2%), pneumonia (24.1%), septic shock (21.1%), UTI (17.7%), and vein thrombosis (14.7%; Supplementary Table S2).

### 3.2 Race-ethnicity

An estimated 61.4% of patients were non-Hispanic White, 23.9% were non-Hispanic Black, 9.5% were Hispanic, and 5.1% were of another race. As shown in Supplementary Table S3, non-Hispanic White patients were most likely to be Medicare beneficiaries (48.6%), have a history of CAD (23.5%), depression (18.6%), GERD (19.3%), ischemic heart disease (21.5%), but less likely to be seen in urban teaching hospitals (77.4%). Non-Hispanic Black patients were younger (median: 49), more likely to be female (55.2%) and treated in the South (54.1%), and less likely to have cirrhosis (4.4%). Hispanic patients were more likely to be male (60.8%), have diabetes (40.0%), obesity (22.8%), and less likely to be treated in the Midwest (7.1%).

**Figure 1:**
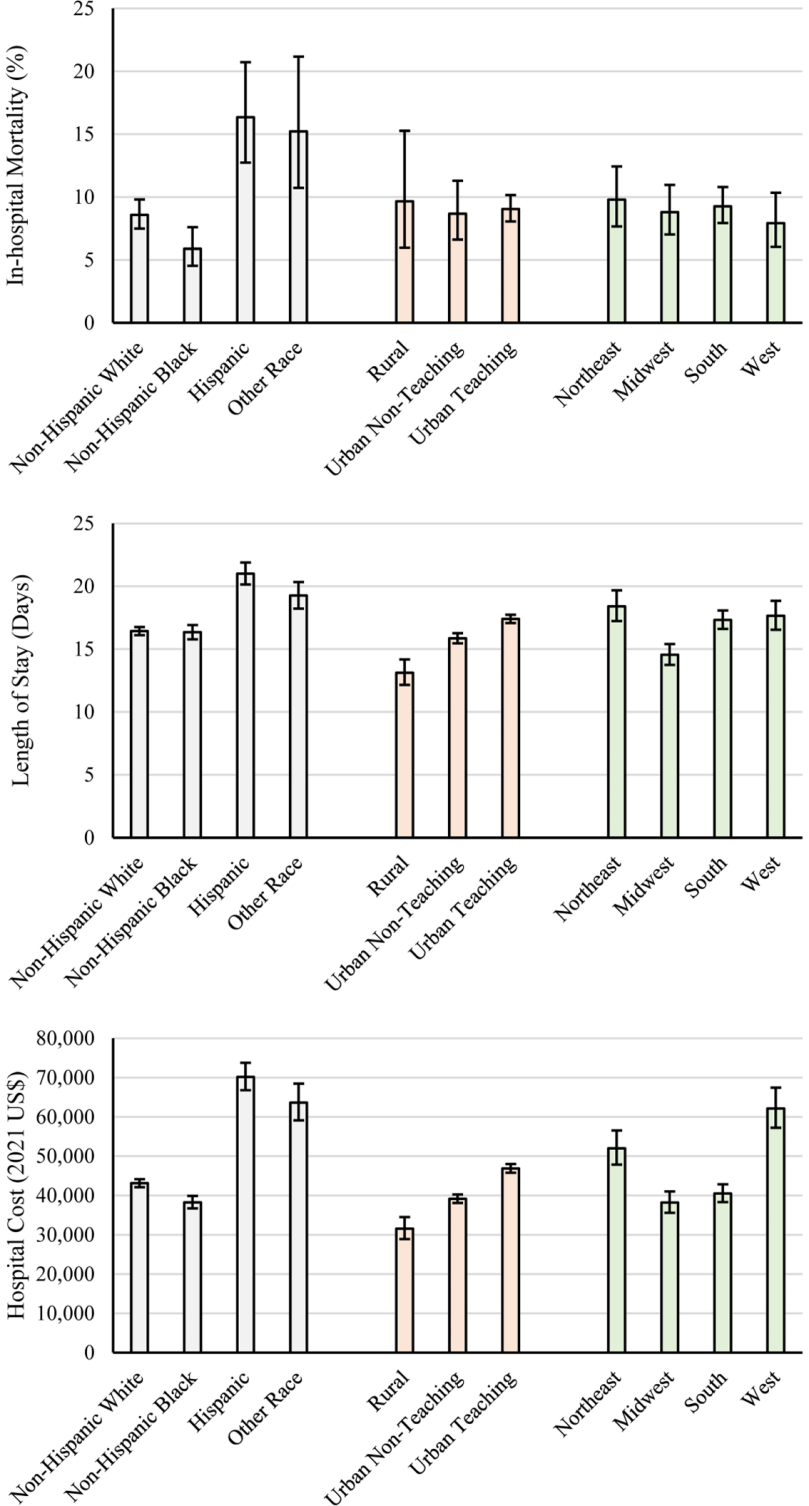
Unadjusted hospital cost (top), length of stay (middle), and inflation-adjusted hospital cost (bottom) by race, location-teaching status, and geographic region. Error bars represent 95% confidence intervals.

Unadjusted in-hospital mortality rates differed by race-ethnicity (*p* < 0.001; Figure 1). After adjusting for demographic and clinical characteristics, Hispanic patients had 95.2% greater odds of death compared to non-Hispanic Blacks (95% CI: 21.1 to 214.7%, *p* = 0.006) and 60.8% greater odds of death compared to non-Hispanic Whites (95% CI: 7.2 to 141.1%, *p* = 0.022; Table 1). Unadjusted length of stay also differed by race-ethnicity (Figure 1). After adjustment, in Hispanics, length of stay was 8.1% longer compared to non-Hispanic Whites (95% CI: 3.7 to 12.7%, *p* < 0.001) and 6.6% longer compared to non-Hispanic Blacks (95% CI: 1.4 to 12.1%, *p* = 0.012; Table 2). Unadjusted hospital cost also differed by race-ethnicity (Figure 1). After adjustment, cost for hospitalizations in which the patient was Hispanic were 29.3% higher than non-Hispanic blacks (95% CI: 22.2 to 36.9%, *p* < 0.001) and 27.0% higher compared to non-Hispanic whites (95% CI: 21.2 to 33.2%, *p* < 0.001; Table 3). In-hospital complications also differed by race-ethnicity with sepsis most likely in Hispanics (70.6%) and least likely in non-Hispanic Blacks (57.7%), septic shock most likely in Hispanics (32.2%) and least likely in non-Hispanic Blacks (15.5%), ARDS most likely in Hispanics (13.9%) and least likely in non-Hispanic Blacks (2.3%), pneumonia most likely in Hispanics (34.9%) and least likely in non-Hispanic Blacks (20.7%), and endocarditis was most likely in non-Hispanic Whites (6.7%) and least likely in non-Hispanic Blacks (2.7%; Supplementary Table S3).

**Table 1:** Unadjusted and adjusted results for all-cause in-hospital mortality.

	Unadjusted		Adjusted	
	OR (95% CI)	*p*	aOR (95% CI)	*p*
Race				
Black vs. White	0.67 (0.49–0.91)	0.011	0.82 (0.58–1.16)	0.269
Hispanic vs. White	2.08 (1.50–2.89)	<0.001	1.61 (1.07–2.41)	0.022
Other vs. White	1.91 (1.25–2.94)	0.003	1.67 (0.98–2.83)	0.059
Black vs. Hispanic	0.32 (0.22–0.48)	<0.001	0.51 (0.32–0.83)	0.006
Black vs. Other	0.35 (0.22–0.56)	<0.001	0.49 (0.28–0.89)	0.018
Hispanic vs. Other	1.09 (0.67–1.77)	0.733	0.96 (0.53–1.77)	0.907
Location-teaching Status				
Rural vs. Urban Teaching	1.07 (0.63–1.84)	0.794	1.16 (0.66–2.05)	0.602
Urban Non-teaching vs. Urban Teaching	0.95 (0.69–1.31)	0.774	0.90 (0.62–1.30)	0.562
Rural vs. Urban Non-teaching	1.13 (0.62–2.05)	0.698	1.30 (0.68–2.47)	0.429
Hospital Region				
Northeast vs. West	1.26 (0.85–1.88)	0.253	1.59 (0.99–2.54)	0.054
Midwest vs. West	1.12 (0.76–1.64)	0.559	1.65 (1.07–2.55)	0.023
South vs. West	1.19 (0.84–1.67)	0.324	1.46 (1.00–2.14)	0.053
Northeast vs. South	1.06 (0.77–1.46)	0.706	1.09 (0.75–1.58)	0.654
Midwest vs. South	0.94 (0.70–1.27)	0.708	1.13 (0.82–1.56)	0.443
Northeast vs. Midwest	1.13 (0.78–1.62)	0.523	0.96 (0.64–1.44)	0.844
Age (per 10 years older)	1.50 (1.39–1.61)	<0.001	1.40 (1.24–1.57)	<0.001
Female vs. Male	0.81 (0.65–1.01)	0.064	1.14 (0.87–1.48)	0.342
Primary Payer				
Medicare vs. Private	1.26 (0.94–1.68)	0.120	0.93 (0.65–1.33)	0.686
Medicaid vs. Private	0.54 (0.37–0.78)	0.001	0.81 (0.53–1.25)	0.348
Other vs. Private	0.61 (0.35–1.07)	0.084	0.57 (0.30–1.10)	0.095
Medicare vs. Other	2.05 (1.21–3.47)	0.007	1.61 (0.85–3.06)	0.141
Medicaid vs. Other	0.88 (0.50–1.55)	0.661	1.42 (0.73–2.73)	0.300
Medicaid vs. Medicare	0.43 (0.31–0.59)	<0.001	0.88 (0.58–1.31)	0.524
Weekend vs. Weekday	1.12 (0.86–1.44)	0.400	0.91 (0.67–1.24)	0.554
Charlson Comorbidity Index	1.14 (1.11–1.18)	<0.001	1.04 (1.00–1.08)	0.050
Individual Comorbidities				
CAD	1.26 (0.97–1.64)	0.089	0.94 (0.69–1.28)	0.680
Depression	0.65 (0.46–0.92)	0.016	0.93 (0.64–1.36)	0.723
Dyslipidemia	1.18 (0.93–1.50)	0.175	0.88 (0.67–1.18)	0.395
Hypertension	1.25 (1.00–1.56)	0.050	0.92 (0.70–1.21)	0.560
Malnutrition	1.04 (0.78–1.37)	0.797	0.82 (0.59–1.12)	0.208
Obesity	1.42 (1.11–1.81)	0.005	1.22 (0.91–1.65)	0.188
Individual Complications				
Sepsis	3.99 (2.93–5.42)	<0.001	1.10 (0.74–1.63)	0.656
Septic shock	8.28 (6.54–10.48)	<0.001	3.73 (2.66–5.23)	<0.001
Severe Sepsis	1.03 (0.69–1.54)	0.883	1.40 (0.85–2.31)	0.182
Acute myocardial infarction	2.65 (1.91–3.67)	<0.001	1.41 (0.94–2.11)	0.099
Cerebral infarction	1.80 (1.09–2.97)	0.021	1.48 (0.81–2.71)	0.206
Cardiogenic shock	1.98 (1.34–2.93)	<0.001	1.24 (0.79–1.97)	0.353
Acute respiratory distress syndrome	8.00 (5.71–11.22)	<0.001	2.62 (1.70–4.03)	<0.001
Disseminated intravascular coagulation	10.50 (6.17–17.86)	<0.001	4.82 (2.52–9.23)	<0.001
Pneumonia	3.93 (3.11–4.96)	<0.001	1.85 (1.39–2.46)	<0.001
Urinary tract infection	1.51 (1.16–1.96)	0.002	1.13 (0.83–1.53)	0.434
Acute kidney injury	4.79 (3.67–6.24)	<0.001	1.93 (1.40–2.68)	<0.001
Acute pancreatitis	1.37 (0.75–2.51)	0.303	1.32 (0.66–2.65)	0.434
Vein thrombosis	1.01 (0.74–1.37)	0.962	0.83 (0.56–1.22)	0.339
Acute and subacute Endocarditis	0.81 (0.48–1.35)	0.414	0.96 (0.50–1.84)	0.907
Pulmonary embolism	1.03 (0.62–1.71)	0.902	1.09 (0.59–2.00)	0.782

OR, odds ratio. aOR, adjusted odds ratio. CI, confidence interval. The reference group is indicated after the “vs.” Odds ratios greater than 1 indicate greater odds of in-hospital mortality.

**Table 2:** Unadjusted and adjusted model results for length of stay in days.

	Unadjusted		Adjusted	
	Ratio (95% CI)	*p*	Ratio (95% CI)	*p*
Race				
Black vs. White	0.99 (0.96–1.03)	0.782	1.01 (0.98–1.05)	0.477
Hispanic vs. White	1.28 (1.22–1.34)	<0.001	1.08 (1.04–1.13)	<0.001
Other vs. White	1.17 (1.10–1.24)	<0.001	1.04 (0.99–1.09)	0.127
Black vs. Hispanic	0.78 (0.74–0.82)	<0.001	0.94 (0.89–0.99)	0.012
Black vs. Other	0.85 (0.79–0.91)	<0.001	0.98 (0.93–1.03)	0.426
Hispanic vs. Other	1.09 (1.02–1.17)	0.014	1.04 (0.99–1.10)	0.141
Location-teaching Status				
Rural vs. Urban Teaching	0.75 (0.70–0.82)	<0.001	0.83 (0.77–0.89)	<0.001
Urban Non-teaching vs. Urban Teaching	0.91 (0.88–0.94)	<0.001	0.93 (0.91–0.95)	<0.001
Rural vs. Urban Non-teaching	0.83 (0.76–0.90)	<0.001	0.89 (0.83–0.96)	0.002
Hospital Region				
Northeast vs. West	1.04 (0.95–1.14)	0.373	1.13 (1.07–1.20)	<0.001
Midwest vs. West	0.82 (0.76–0.90)	<0.001	0.87 (0.83–0.91)	<0.001
South vs. West	0.98 (0.91–1.06)	0.640	1.02 (1.01–1.03)	<0.001
Northeast vs. South	1.06 (0.98–1.15)	0.130	1.10 (1.04–1.17)	0.001
Midwest vs. South	0.84 (0.78–0.90)	<0.001	0.85 (0.81–0.89)	<0.001
Northeast vs. Midwest	1.27 (1.16–1.38)	<0.001	1.30 (1.21–1.40)	<0.001
Age (per 10 years older)	1.01 (1.00–1.02)	0.006	0.99 (0.98–1.00)	0.051
Female vs. Male	0.83 (0.81–0.86)	<0.001	0.89 (0.86–0.91)	<0.001
Primary Payer				
Medicare vs. Private	1.01 (0.97–1.05)	0.704	0.99 (0.95–1.03)	0.514
Medicaid vs. Private	1.19 (1.13–1.25)	<0.001	1.21 (1.15–1.26)	<0.001
Other vs. Private	1.23 (1.16–1.31)	<0.001	1.16 (1.11–1.22)	<0.001
Medicare vs. Other	0.82 (0.78–0.86)	<0.001	0.85 (0.81–0.89)	<0.001
Medicaid vs. Other	0.96 (0.91–1.02)	0.159	1.04 (0.99–1.09)	0.135
Medicaid vs. Medicare	1.18 (1.13–1.22)	<0.001	1.22 (1.17–1.27)	<0.001
Weekend vs. Weekday	1.01 (0.97–1.04)	0.791	1.01 (0.98–1.04)	0.662
Charlson Comorbidity Index	1.07 (1.06–1.07)	<0.001	1.05 (1.04–1.05)	<0.001
Individual Comorbidities				
CAD	0.96 (0.93–0.99)	0.017	0.94 (0.91–0.97)	<0.001
Depression	0.99 (0.95–1.04)	0.646	1.09 (1.05–1.13)	<0.001
Dyslipidemia	0.93 (0.90–0.97)	<0.001	0.96 (0.93–1.00)	0.036
Hypertension	0.96 (0.94–0.99)	0.017	0.96 (0.93–0.99)	0.004
Malnutrition	1.39 (1.33–1.45)	<0.001	1.32 (1.27–1.37)	<0.001
Obesity	1.08 (1.04–1.12)	<0.001	1.06 (1.03–1.10)	<0.001
Individual Complications				
Sepsis	1.51 (1.46–1.56)	<0.001	1.23 (1.19–1.28)	<0.001
Septic shock	1.51 (1.46–1.56)	<0.001	1.01 (0.98–1.05)	0.494
Severe Sepsis	1.17 (1.11–1.24)	<0.001	1.00 (0.95–1.05)	0.869
Acute myocardial infarction	1.77 (1.59–1.97)	<0.001	1.48 (1.36–1.62)	<0.001
Cerebral infarction	1.26 (1.19–1.32)	<0.001	0.99 (0.94–1.04)	0.645
Cardiogenic shock	1.46 (1.36–1.57)	<0.001	1.26 (1.16–1.36)	<0.001
Acute respiratory distress syndrome	1.28 (1.21–1.35)	<0.001	1.11 (1.05–1.18)	0.001
Disseminated intravascular coagulation	1.67 (1.57–1.78)	<0.001	1.13 (1.07–1.20)	<0.001
Pneumonia	1.47 (1.37–1.58)	<0.001	1.02 (0.95–1.10)	0.557
Urinary tract infection	1.59 (1.54–1.65)	<0.001	1.31 (1.26–1.35)	<0.001
Acute kidney injury	1.20 (1.15–1.25)	<0.001	1.12 (1.08–1.16)	<0.001
Acute pancreatitis	0.81 (0.76–0.86)	<0.001	0.86 (0.81–0.91)	<0.001
Vein thrombosis	1.01 (0.91–1.11)	0.908	1.13 (1.03–1.24)	0.008
Acute and subacute Endocarditis	1.47 (1.43–1.51)	<0.001	1.22 (1.18–1.25)	<0.001
Pulmonary embolism	1.27 (1.18–1.36)	<0.001	1.17 (1.09–1.26)	<0.001

CI, confidence interval. The reference group is indicated after the “vs.” Ratios greater than 1 indicate longer length of stay.

**Table 3:** Unadjusted and adjusted model results for inflation-adjusted hospital cost (2021 US$).

	Unadjusted		Adjusted	
	Ratio (95% CI)	*p*	Ratio (95% CI)	*p*
Race				
Black vs. White	0.89 (0.85–0.93)	<0.001	0.98 (0.94–1.03)	0.418
Hispanic vs. White	1.63 (1.54–1.72)	<0.001	1.27 (1.21–1.33)	<0.001
Other vs. White	1.48 (1.37–1.59)	<0.001	1.18 (1.11–1.26)	<0.001
Black vs. Hispanic	0.55 (0.51–0.58)	<0.001	0.77 (0.73–0.82)	<0.001
Black vs. Other	0.60 (0.55–0.66)	<0.001	0.83 (0.78–0.89)	<0.001
Hispanic vs. Other	1.10 (1.01–1.20)	0.027	1.07 (1.00–1.15)	0.042
Location-teaching Status				
Rural vs. Urban Teaching	0.67 (0.61–0.74)	<0.001	0.78 (0.73–0.85)	<0.001
Urban Non-teaching vs. Urban Teaching	0.84 (0.81–0.87)	<0.001	0.86 (0.84–0.88)	<0.001
Rural vs. Urban Non-teaching	0.81 (0.73–0.88)	<0.001	0.91 (0.85–0.98)	0.016
Hospital Region				
Northeast vs. West	0.84 (0.74–0.94)	0.003	0.92 (0.86–0.98)	0.014
Midwest vs. West	0.62 (0.55–0.69)	<0.001	0.66 (0.63–0.7)	<0.001
South vs. West	0.65 (0.59–0.72)	<0.001	0.7 (0.69–0.71)	<0.001
Northeast vs. South	1.28 (1.16–1.42)	<0.001	1.31 (1.23–1.41)	<0.001
Midwest vs. South	0.94 (0.86–1.03)	0.200	0.95 (0.9–1.01)	0.081
Northeast vs. Midwest	1.36 (1.22–1.52)	<0.001	1.38 (1.26–1.51)	<0.001
Age (per 10 years older)	1.06 (1.05–1.07)	<0.001	1.01 (1.00–1.03)	0.065
Female vs. Male	0.73 (0.70–0.75)	<0.001	0.82 (0.8–0.85)	<0.001
Primary Payer				
Medicare vs. Private	0.91 (0.86–0.96)	0.001	0.88 (0.84–0.92)	<0.001
Medicaid vs. Private	1.00 (0.94–1.06)	0.946	1.04 (0.99–1.09)	0.104
Other vs. Private	1.00 (0.94–1.07)	0.898	0.98 (0.93–1.04)	0.575
Medicare vs. Other	0.90 (0.85–0.96)	0.001	0.89 (0.84–0.94)	<0.001
Medicaid vs. Other	0.99 (0.93–1.06)	0.839	1.06 (1.00–1.12)	0.039
Medicaid vs. Medicare	1.10 (1.05–1.15)	<0.001	1.19 (1.14–1.25)	<0.001
Weekend vs. Weekday	1.03 (0.98–1.07)	0.256	0.98 (0.95–1.02)	0.391
Charlson Comorbidity Index	1.08 (1.08–1.09)	<0.001	1.05 (1.04–1.05)	<0.001
Individual Comorbidities				
CAD	1.05 (1.01–1.10)	0.026	0.96 (0.92–1.00)	0.046
Depression	0.89 (0.85–0.94)	<0.001	1.04 (0.99–1.08)	0.138
Dyslipidemia	0.97 (0.93–1.02)	0.188	0.96 (0.92–1.00)	0.053
Hypertension	0.96 (0.92–0.99)	0.021	0.94 (0.91–0.97)	<0.001
Malnutrition	1.48 (1.41–1.56)	<0.001	1.37 (1.31–1.43)	<0.001
Obesity	1.21 (1.16–1.26)	<0.001	1.14 (1.10–1.18)	<0.001
Individual Complications				
Sepsis	1.69 (1.63–1.76)	<0.001	1.19 (1.14–1.24)	<0.001
Septic shock	2.12 (2.04–2.20)	<0.001	1.22 (1.17–1.27)	<0.001
Severe Sepsis	1.19 (1.11–1.27)	<0.001	1.05 (0.99–1.11)	0.098
Acute myocardial infarction	2.16 (1.90–2.45)	<0.001	1.59 (1.45–1.75)	<0.001
Cerebral infarction	1.70 (1.60–1.81)	<0.001	1.11 (1.05–1.17)	<0.001
Cardiogenic shock	1.78 (1.64–1.94)	<0.001	1.47 (1.34–1.61)	<0.001
Acute respiratory distress syndrome	2.06 (1.91–2.23)	<0.001	1.57 (1.44–1.71)	<0.001
Disseminated intravascular coagulation	2.75 (2.56–2.96)	<0.001	1.40 (1.31–1.49)	<0.001
Pneumonia	2.27 (2.08–2.48)	<0.001	1.28 (1.21–1.35)	<0.001
Urinary tract infection	2.01 (1.93–2.09)	<0.001	1.45 (1.39–1.51)	<0.001
Acute kidney injury	1.16 (1.11–1.21)	<0.001	1.06 (1.02–1.10)	0.005
Acute pancreatitis	0.68 (0.64–0.72)	<0.001	0.78 (0.73–0.83)	<0.001
Vein thrombosis	0.89 (0.81–0.99)	0.031	1.11 (1.00–1.22)	0.041
Acute and subacute Endocarditis	1.78 (1.71–1.84)	<0.001	1.27 (1.22–1.31)	<0.001
Pulmonary embolism	1.43 (1.32–1.55)	<0.001	1.28 (1.18–1.38)	<0.001

CI, confidence interval. The reference group is indicated after the “vs.” Ratios greater than 1 indicate higher inflation-adjusted hospital cost.

### 3.3 Location-teaching status

Regarding location-teaching status, 80.1% occurred in urban teaching hospitals, 15.2% occurred in urban non-teaching hospitals, and 4.7% occurred in rural hospitals. As shown in Supplementary Table S4: hospitalizations in urban teaching hospitals were more likely to include non-Hispanic Black patients (25.4%), be in the Northeast (18.2%), and be less likely to include a patient with hypertension (47.3%). Hospitalizations in urban non-teaching hospitals were more likely to be in the South (54.1%). Hospitalizations in rural hospitals were more likely to include non-Hispanic White patients (84.2%) and be in the Midwest (30.5%).

Unadjusted in-hospital morality rates were statistically similar across location-teaching status (Figure 1); no adjusted differences were indicated (Table 1). Unadjusted length of stay differed by location-teaching status (Figure 1). After adjustment, urban teaching hospitals had 20.5% longer length of stay compared to rural hospitals (95% CI: 12.4 to 29.2%, *p* < 0.001) and 7.6% longer length of stay compared to urban non-teaching hospitals (95% CI: 4.8 to 10.4%, *p* < 0.001); urban non-teaching hospitals had 12.0% longer length of stay compared to rural hospitals (95% CI: 4.4 to 20.2%, *p* = 0.002; Table 2). Unadjusted hospital cost also differed by location-teaching status (Figure 1). After adjustment, hospital cost in urban teaching hospitals was 27.5% higher compared to rural hospitals (95% CI: 18.3 to 37.5%, *p* < 0.001) and 16.2% higher compared to urban non-teaching hospitals (95% CI: 13.1 to 19.5%, *p* < 0.001); cost was 9.7% higher in urban non-teaching hospitals compared to rural hospitals (95% CI: 1.8 to 18.3%, *p* = 0.016; Table 3). In-hospital complications also differed by location-teaching status with severe sepsis most likely in urban non-teaching hospitals (10.1%) and least likely in rural hospitals (6.4%) and vein thrombosis most likely in urban teaching hospitals (15.5%) and least likely in rural hospitals (8.0%; Supplementary Table S4).

### 3.4 Geographic region of the United States

For geographic region of the United States, 44.1% of hospitalizations occurred in the South, 22.4% occurred in the Midwest, 17.1% occurred in the West, and 16.4% occurred in the Northeast. As shown in Supplementary Table S5, Hospitalizations in the South had the highest rate non-Hispanic black patients (29.1%). The West had the highest rate of hospitalizations including Medicaid beneficiaries (34.1%), Hispanic patients (18.9%), and patients with alcohol dependence (8.6%). The Northeast had the highest rate of hospitalizations to urban teaching hospitals (88.8%). The Midwest had the highest rate of hospitalizations including hypertension (51.9%), Medicare beneficiaries (49.0%), dyslipidemia (33.7%), obesity (29.6%), patients with CAD (26.9%), malnutrition (26.5%), ischemic heart disease (24.2%), depression (22.6%), GERD (22.3%), CKD (19.1%), and malignancy (9.9%).

Unadjusted in-hospital morality rates were statistically similar across geographic regions (Figure 1); however, after adjustment, odds of in-hospital mortality were 65.5% higher in the Midwest compared to the West (95% CI: 7.4 to 155.1%, *p* = 0.023; Table 1). Unadjusted length of stay differed by geographic region (Figure 1). After adjustment, when compared to hospitalizations in the Midwest, hospitalizations were 30.4% longer in the Northeast (95% CI: 21.1 to 40.4%, *p* < 0.001), 18.2% longer in the South (95% CI: 12.6 to 24.0%, *p* < 0.001), and 15.4% longer in the West (95% CI: 10.0 to 21.0%, *p* < 0.001); hospitalizations in the Northeast were also 13.0% longer compared to the West (95% CI: 6.7 to 19.8%, *p* < 0.001) and 10.3% longer compared to the South (95% CI: 4.1 to 17.0%, *p* = 0.001; Table 2). Unadjusted hospital cost differed by geographic region (Figure 1). After adjustment, hospitalizations in the West were 50.6% higher compared to the Midwest (95% CI: 42.2 to 59.5%, *p* < 0.001), 43.2% higher compared to the South (95% CI: 41.6% to 44.75, *p* < 0.001), and 9.1% higher compared to the Northeast (1.8 to 16.9%, *p* = 0.014); hospital costs for hospitalizations in the Northeast were 38.1% higher compared to the Midwest (95% CI: 26.4 to 50.8%, *p* < 0.001) and 31.2% higher compared to the South (95% CI: 22.5 to 40.6%, *p* < 0.001; Table 3). In-hospital complications also differed by geographic region with sepsis and septic shock most likely in the West (65.7 and 24.4%, respectively) and least likely in the Midwest (57.7 and 17.9%, respectively; Supplementary Table S5).

## 4 Discussion

CLABSI are serious complications of central venous catheters that increase hospital length of stay, costs, morbidity, and mortality. Although recent interventions have led to a decrease in incidence, CLABSI remains a serious burden to patients and the United States healthcare system. The existing literature largely focuses on the differing rates of CLABSI among hospital settings and patient populations; far less has been discovered about the impact of the problem as measured by mortality, cost, and length of stay. Numerous comorbid conditions predispose a patient to CLABSI, including diabetes, hypertension, depression, and obesity, and the prevalence of these conditions may vary across populations. Our model accounts for this to reveal differences in mortality, cost, and length of stay which can be attributed to demographic characteristics. Our analysis discovered that race-ethnicity, hospital location-teaching status, and hospital region were all significantly associated with mortality, cost, and length of stay for adult patients with CLABSI.

First, Hispanic patients had worse outcomes compared to other ethnic/racial groups while non-Hispanic Black patients had comparable or improved outcomes compared to other racial groups. Our study found no significant difference in hospital death rates or hospital costs between non-Hispanic Black and non-Hispanic White patients. However, non-Hispanic Black patients had approximately 50% lower odds of death compared to both Hispanic and other racial groups, 6% shorter LOS compared to Hispanic patients, and approximately 20% lower cost of hospitalization compared to Hispanic patient and other racial groups. This may be explained by varied prevalence of comorbid conditions and outcomes which may increase the lethality of CLABSI. For instance, our data reveals a significantly higher prevalence of complicated diabetes mellitus and peripheral vascular disease among Hispanic patients. Additionally, sepsis, a major driver of mortality and LOS, was most prevalent among Hispanic patients and least prevalent among non-Hispanic Black patients. The underlying disparities in these chronic diseases may be tied to social determinants of health and should be more thoroughly explored.

A 2018–2021 retrospective study at a single institution found that rates of CLABSI were significantly higher in non-Hispanic Black and Hispanic/Latino patients than in non-Hispanic White patients (9). However, this study did not account for comorbid conditions which may be present at different rates in different racial groups, and therefore is unable to conclude that the differences were attributable to race alone. Another retrospective study from the Tennessee Hospital Discharge Data System from 2018 to 2021 found that Black patients had a 79% higher risk of contracting CLABSI after controlling for Charlson comorbidity index, age group, and social vulnerability index (10). In addition, our study revealed that Hispanic patients had longer LOS, higher mortality rate, and higher cost of hospitalization compared to non-Hispanic White patients.

One retrospective review of 79,000 adult inpatients with Medicare between 2009 and 2011 adjusted for a set of eight comorbid conditions and found that Hispanic adults had higher rates of healthcare-associated infections overall. However, this consisted largely of catheter-associated urinary tract infections and ventilator-associated pneumonia; the Hispanic group had the lowest odds of CLABSI (1.1), compared with non-Hispanic Black (1.7) and non-Hispanic White (1.2), and Asian (2.3) racial-ethnic groups (19). This investigation was limited to hospitalizations for acute cardiovascular disease, pneumonia, and surgical care, whereas our study included all hospitalizations and focused on outcomes instead of CLABSI rates. Taken together though, our findings and that of previous literature points to an association between Hispanic ethnicity and CLABSI that warrants further investigation.

One possible explanation for disparities based upon race is that despite efforts for quality care, under stress or fatigue, healthcare workers might resort to cognitive shortcuts, making them vulnerable to unconscious biases. Minority racial groups are also more vulnerable to language barriers which may result in miscommunication about appropriate catheter manipulation by patients, delayed symptom reporting, and mistrust between patients and providers which culminates to make them more vulnerable to CLABSI. Further, prior research indicates that the racial disparities in CLABSI rates are partly influenced by the hospitals where minority patients receive care. Hospitals serving a higher proportion of minority patients may have less funding, fewer resources, and lower staffing levels, which can contribute to higher infection rates (19).

Second, rural hospitals had shorter length of stay and cost compared to urban teaching and non-teaching hospitals. Urban non-teaching hospitals also had shorter length of stay and cost compared to urban teaching hospitals. Severe sepsis and deep vein thrombosis were both more likely in urban teaching hospitals and least likely in rural hospitals, though there was no significant difference in mortality rates between the three locations. One study in California found no relationship between location and CLABSI rates (12), but this otherwise has not been well studied in existing literature. More generally, literature mentions how rural hospitals have less emphasis on infection prevention (20). A study of 77 small rural hospitals in the Western United States found that almost all infection disease professionals employed in rural hospitals also held other job responsibilities and dedicated an average of 25% of the week to infection control (21). One possible explanation for our findings of superior outcomes in rural hospitals may be that sicker patients, those more likely to have severe sepsis, thrombotic complications, and lengthy and expensive stays have already been transferred to larger hospitals in urban settings by the time they develop a CLABSI.

Lastly, LOS was longer in the Northeast compared to the West and South, and shorter in the Midwest compared to the West and South. Additionally, costs were lower in the Midwest and South compared to the West, whereas the Northeast had higher costs compared to all other regions. We found no significant difference in hospital mortality rates between hospital regions in the West, Midwest, South, and Northeast. It is unclear why these differences exist as there is a dearth of research on this topic. Longer LOS in Northeast could be due to higher number of teaching hospitals, which are associated with longer LOS (22). Lower costs in the Midwest and the South could be due to lower cost of living or variations in reimbursement rates that lead to higher charges in the Northeast. Lastly, the similarity in mortality rates could be due to standardization of medical care, adherence to clinical guidelines, and national healthcare regulations that ensure similar hospital mortality rates across regions.

This study has several limitations inherent to using the National Inpatient Sample (NIS) for CLABSI epidemiology. The NIS does not stratify data by ICU status, potentially obscuring the higher risk and incidence of CLABSI in these settings. Conflation of race and ethnicity categories, as well as the combined other race category, may mask specific disparities. Coding challenges in distinguishing primary from secondary infections might have led to the underreporting of CLABSI cases. The NIS also does not distinguish between rural teaching and non-teaching hospitals. Additionally, determining if infections were present on admission versus hospital-acquired remains difficult, and inaccuracies in medical charting can further compromise data reliability.

In conclusion, this study highlights how race, hospital geographical location, and region are associated with length of stay, costs, and mortality rates in adult patients with CLABSI. These findings underscore the need for targeted interventions and further research to address the underlying causes of these disparities and improve CLABSI management across diverse patient populations and hospital settings.

## Data Availability

Publicly available datasets were analyzed in this study. This data can be found at: https://hcup-us.ahrq.gov/databases.jsp.
