# Supplementary material for: Demographic-based disparities in outcomes for adults with central line-associated bloodstream infections in the United States: a National Inpatient Sample database study (2016–2020)
Source: Front Med (Lausanne). 2024 Oct 11;11:1469522. doi: 10.3389/fmed.2024.1469522 (PMC11502380; doi:10.3389/fmed.2024.1469522)
Supplement: Supplementary file 1 [file Table_1.DOCX]

**Supplemental Content**

**Table S1.** ICD-10 diagnosis and procedure codes 2

**Table S2.** Demographic and clinical characteristics of the overall hospitalization cohort 10

**Table S3.** Demographic and clinical characteristics stratified by race/ethnicity 13

**Table S4.** Demographic and clinical characteristics stratified by location-teaching status 17

**Table S5.** Demographic and clinical characteristics stratified by geographic region 21

**Table S1.** ICD-10 diagnosis and procedure codes

| **Diagnosis** | **ICD-10-CM** | **Elixhauser** |
| --- | --- | --- |
| CLABSI | T80211A |  |
| **Exclusion Criteria** |  |  |
| Cancer | C, D47Z2, D47Z9 |  |
| Immunosuppressed | B20, B59, C802, C888, C9440, C9441, C9442, C946, D4622, D4701, D4702, D4709, D471, D479, D47Z1, D47Z2, D47Z9, D6109, D61810, D61811, D61818, D700, D701, D702, D704, D708, D709, D71, D720, D72810, D72818, D72819, D7381, D7581, D761, D762, D763, D800, D801, D802, D803, D804, D805, D806, D807, D808, D809, D810, D811, D812, D8130, D8131, D8132, D8139, D814, D816, D817, D8182, D8189, D819, D820, D821, D822, D823, D824, D828, D829, D830, D831, D832, D838, D839, D840, D841, D848, D8481, D84821, D84822, D8489, D849, D893, D89810, D89811, D89812, D89813, D8982, D8989, D899, E40, E41, E42, E43, I120, I1311, I132, K912, N185, N186, T8600, T8601, T8602, T8603, T8609, T8610, T8611, T8612, T8613, T8619, T8620, T8621, T8622, T8623, T86290, T86298, T8630, T8631, T8632, T8633, T8639, T8640, T8641, T8642, T8643, T8649, T865, T86810, T86811, T86812, T86818, T86819, T86850, T86851, T86852, T86858, T86859, T86890, T86891, T86892, T86898, T86899, T8690, T8691, T8692, T8693, T8699, Z4821, Z4822, Z4823, Z4824, Z48280, Z48288, Z48290, Z48298, Z4901, Z4902, Z4931, Z4932, Z940, Z941, Z942, Z943, Z944, Z9481, Z9482, Z9483, Z9484, Z9489, Z992 |  |
| **Covariates** |  |  |
| Asplenia | Q8901 |  |
| Burn | T2000XA, T20011A, T20012A, T20019A, T2002XA, T2003XA, T2004XA, T2005XA, T2006XA, T2007XA, T2009XA, T2010XA, T20111A, T20112A, T20119A, T2012XA, T2013XA, T2014XA, T2015XA, T2016XA, T2017XA, T2019XA, T2020XA, T20211A, T20212A, T20219A, T2022XA, T2023XA, T2024XA, T2025XA, T2026XA, T2027XA, T2029XA, T2030XA, T20311A, T20312A, T20319A, T2032XA, T2033XA, T2034XA, T2035XA, T2036XA, T2037XA, T2039XA, T2100XA, T2101XA, T2102XA, T2103XA, T2104XA, T2105XA, T2106XA, T2107XA, T2109XA, T2110XA, T2111XA, T2112XA, T2113XA, T2114XA, T2115XA, T2116XA, T2117XA, T2119XA, T2120XA, T2121XA, T2122XA, T2123XA, T2124XA, T2125XA, T2126XA, T2127XA, T2129XA, T2130XA, T2131XA, T2132XA, T2133XA, T2134XA, T2135XA, T2136XA, T2137XA, T2139XA, T2200XA, T22011A, T22012A, T22019A, T22021A, T22022A, T22029A, T22031A, T22032A, T22039A, T22041A, T22042A, T22049A, T22051A, T22052A, T22059A, T22061A, T22062A, T22069A, T22091A, T22092A, T22099A, T2210XA, T22111A, T22112A, T22119A, T22121A, T22122A, T22129A, T22131A, T22132A, T22139A, T22141A, T22142A, T22149A, T22151A, T22152A, T22159A, T22161A, T22162A, T22169A, T22191A, T22192A, T22199A, T2220XA, T22211A, T22212A, T22219A, T22221A, T22222A, T22229A, T22231A, T22232A, T22239A, T22241A, T22242A, T22249A, T22251A, T22252A, T22259A, T22261A, T22262A, T22269A, T22291A, T22292A, T22299A, T2230XA, T22311A, T22312A, T22319A, T22321A, T22322A, T22329A, T22331A, T22332A, T22339A, T22341A, T22342A, T22349A, T22351A, T22352A, T22359A, T22361A, T22362A, T22369A, T22391A, T22392A, T22399A, T23001A, T23002A, T23009A, T23011A, T23012A, T23019A, T23021A, T23022A, T23029A, T23031A, T23032A, T23039A, T23041A, T23042A, T23049A, T23051A, T23052A, T23059A, T23061A, T23062A, T23069A, T23071A, T23072A, T23079A, T23079A, T23092A, T23099A, T23101A, T23102A, T23109A, T23111A, T23112A, T23119A, T23121A, T23122A, T23129A, T23131A, T23132A, T23139A, T23141A, T23142A, T23149A, T23151A, T23152A, T23159A, T23161A, T23162A, T23169A, T23171A, T23172A, T23179A, T23191A, T23192A, T23199A, T23201A, T23202A, T23209A, T23211A, T23212A, T23219A, T23221A, T23222A, T23229A, T23231A, T23232A, T23239A, T23241A, T23242A, T23249A, T23251A, T23252A, T23259A, T23261A, T23262A, T23269A, T23271A, T23272A, T23279A, T23291A, T23292A, T23299A, T23301A, T23302A, T23309A, T23311A, T23312A, T23319A, T23321A, T23322A, T23329A, T23331A, T23332A, T23339A, T23341A, T23342A, T23349A, T23351A, T23352A, T23359A, T23361A, T23362A, T23369A, T23371A, T23372A, T23379A, T23391A, T23392A, T23399A, T24001A, T24002A, T24009A, T24011A, T24012A, T24019A, T24021A, T24022A, T24029A, T24031A, T24032A, T24039A, T24091A, T24092A, T24099A, T24101A, T24102A, T24109A, T24111A, T24112A, T24119A, T24121A, T24122A, T24129A, T24131A, T24132A, T24139A, T24191A, T24192A, T24199A, T24201A, T24202A, T24209A, T24211A, T24212A, T24219A, T24221A, T24222A, T24229A, T24231A, T24232A, T24239A, T24291A, T24292A, T24299A, T24301A, T24302A, T24309A, T24311A, T24312A, T24319A, T24321A, T24322A, T24329A, T24331A, T24332A, T24339A, T24391A, T24392A, T24399A, T25011A, T25012A, T25019A, T25021A, T25022A, T25029A, T25031A, T25032A, T25039A, T25091A, T25092A, T25099A, T25111A, T25112A, T25119A, T25121A, T25122A, T25129A, T25131A, T25132A, T25139A, T25191A, T25192A, T25199A, T25211A, T25212A, T25219A, T25221A, T25222A, T25229A, T25231A, T25232A, T25239A, T25291A, T25292A, T25299A, T25311A, T25312A, T25319A, T25321A, T25322A, T25329A, T25331A, T25332A, T25339A, T25391A, T25392A, T25399A |  |
| CAD | I24, I25, I200, I519 |  |
| History of Cancer | Z8500, Z8501, Z85020, Z85028, Z85030, Z85038, Z85040, Z85048,Z8505, Z85060, Z85068, Z8507, Z8509, Z85110, Z85118, Z8512,Z8520, Z8521, Z8522, Z85230, Z85238, Z8529, Z853, Z8540,Z8541, Z8542, Z8543, Z8544, Z8545, Z8546, Z8547, Z8548, Z8549,Z855, Z8551, Z85520, Z85528, Z8553, Z8554, Z8559, Z856, Z8571,Z8572, Z8579, Z85810, Z85818, Z85819, Z85820, Z85821, Z85828,Z85830, Z85831, Z85840, Z85841, Z85848, Z85850, Z85858, Z8589, Z859 |  |
| Cerebral palsy | G800, G801, G802, G803, G804, G808, G809 |  |
| Chronic corticosteroids | Z7952, Z7951 |  |
| Liver Cirrhosis |  | CM_LIVER |
| CKD |  | CM_RENLFAIL |
| Congestive Heart Failure |  | CM_CHF |
| Crohn's Disease | K50 |  |
| Cystic fibrosis | E84 |  |
| Dementia | F0150, F01511, F01518, F0152, F0153, F0154, F01A0, F01A11, F01A18, F01A2, F01A3, F01A4, F01B0, F01B11, F01B18, F01B2, F01B3, F01B4, F01C0, F01C11, F01C18, F01C2, F01C3, F01C4, F0280, F02811, F02818, F0282, F0283, F0284, F02A0, F02A11, F02A18, F02A2, F02A3, F02A4, F02B0, F02B11, F02B18, F02B2, F02B3, F02B4, F02C0, F02C11, F02C18, F02C2, F02C3, F02C4, F0390, F03911, F03918, F0392, F0393, F0394, F03A0, F03A11, F03A18, F03A2, F03A3, F03A4, F03B0, F03B11, F03B18, F03B2, F03B3, F03B4, F03C0, F03C11, F03C18, F03C2, F03C3, F03C4, F0670, F0671, F068 |  |
| Depression |  | CM_DEPRESS |
| Diabetes Mellitus |  | CM_DM, CM_DMCX |
| Dyslipidemia | E780, E781, E782, E783, E784, E785 |  |
| Gallstones | K8018, K8020, K8011, K8010, K8000, K8021, K8060, K8001, K8019, K8061 |  |
| GERD | K2100, K2101, K219 |  |
| Hemiplegia | G8100, G8101, G8102, G8103, G8104, G8110, G8111, G8112, G8113, G8114, G8190, G8191, G8192, G81.93, G8194 |  |
| Hemodialysis | Z992 |  |
| HIV/AIDS | B20 |  |
| Hypertension |  | CM_HTN_C |
| IBS | K580, K581, K582, K588, K589 |  |
| Ischemic heart disease | I200, I201, I202, I2081, I2089, I209, I240, I2481, I2489, I249, I2510, I25110, I25111, I25I12, I25118, I25119, I253, I2541, I2542, I255, I256, I25700, I25701, I25702, I25708, I25709, I25710, I25711, I25712, I25718, I25719, I25720, I25721, I25722, I25728, I25729, I25730, I25731, I25732, I25738, I25739, I25750, I25751, I25752, I25758, I25759, I25760, I25761, I25762, I25768, I25769, I25790, I25791, I25792, I25798, I25799, I25810, I25811, I25812, I2582, I2583, I2584, I2585, I2589, I259 |  |
| Left Ventricular Systolic Dysfunction | I501 |  |
| Malnutrition | E46, E440, E441, E43 |  |
| Mechanical ventilation | Z9911 |  |
| History of MI | I252 |  |
| Nephrolithiasis | N200, N201, N202, N209, N210, N211, N218, N219 |  |
| Obesity |  | CM_OBESE |
| Paraplegia | G822 |  |
| Pulmonary hypertension |  | CM_PULMCIRC |
| PVD |  | CM_PERIVASC |
| Quadriplegia | G825 |  |
| Sickle cell | D57 |  |
| Spinal cord injury | S341, S14109, S24109 |  |
| History of Stroke/ TIA | Z8673 |  |
| History of Transplant | Z949 |  |
| Ulcerative colitis | K51 |  |
| **Complications** |  |  |
| Sepsis | A400, A401, A403, A408, A409, A4101, A4102, A411, A412, A413, A414, A4150, A4151, A4152, A4153, A4154, A4159, A4181, A4189, A419, A427, A227, B377, A267, A282, A5486, A327, A241, A392, A207, A217, A483 |  |
| Septic shock | R6521 |  |
| Severe Sepsis | R6520 |  |
| Resistance to unspecified antimicrobial drugs | Z1630 |  |
| Mild cognitive impairment | G3184 |  |
| Polyneuropathy | G600, G601, G602, G603, G608, G609, G610, G611, G6181, G6182, G6189, G619, G620, G621, G622, G6281, G6282, G6289, G629, G63 |  |
| Acute MI | I2101, I2102, I2109, I2111, I2119, I2121, I2129, I213, I214, I21A1, I21A9, I21B |  |
| Cerebral infarction | I6300, I63011, I63012, I63013, I63019, I6302, I63031, I63032, I63033, I63039, I6309, I6310, I63111, I63112, I63119, I6312, I63131, I63132, I63133, I63139, I6319, I6320, I63211, I63212, I63213, I63219, I6322, I63231, I63232, I63233, I63239, I6329, I6330, I63311, I63312, I63313, I63319, I63321, I63322, I63329, I63331, I63332, I63333, I63339, I63341, I63342, I63343, I63349, I6339, I6340, I63411, I63412, I63413, I63419, I63421, I63422, I63423, I63429, I63431, I63432, I63433, I63439, I63441, I63442, I63443, I63449, I6349, I6350, I63511, I63512, I63513, I63519, I63521, I63522, I63523, I63529, I63531, I63532, I63533, I63539, I63541, I63542, I63543, I63549, I6359, I636, I6381, I6389, I639 |  |
| Cardiogenic shock | R570 |  |
| Acute respiratory distress syndrome | J80 |  |
| Disseminated intravascular coagulation | D65 |  |
| Infective myocarditis | I400 |  |
| Pneumonia | J120, J121, J122, J123, J1281, J1282, J1289, J13, J14, J150, J151, J15211, J15212, J1529, J153, J154, J155, J1561, J1569, J157, J158, J159, J189, J160 |  |
| Urinary tract infection | N390 |  |
| Osteomyelitis | M8600, M86011, M86012, M86019, M86021, M86022, M86029, M86031, M86032, M86039, M86041, M86042, M86049, M86051, M86052, M86059, M86061, M86062, M86069, M86071, M86072, M86079, M8608, M8609, M8610, M86111, M86112, M86119, M86121, M86122, M86129, M86131, M86132, M86139, M86141, M86142, M86149, M86151, M86152, M86159, M86161, M86162, M86169, M86171, M86172, M86179, M8618, M8619, M8620, M86211, M86212, M86219, M86221, M86222, M86229, M86231, M86232, M86239, M86241, M86242, M86249, M86251, M86252, M86259, M86261, M86262, M86269, M86271, M86272, M86279, M8628, M8629, M8630, M86311, M86312, M86319, M86321, M86322, M86329, M86331, M86332, M86339, M86341, M86342, M86349, M86351, M86352, M86359, M86361, M86362, M86369, M86371, M86372, M86379, M8638, M8639, M8640, M86411, M86412, M86419, M86421, M86422, M86429, M86431, M86432, M86439, M86441, M86442, M86449, M86451, M86452, M86459, M86461, M86462, M86469, M86471, M86472, M86479, M8648, M8649, M8650, M86511, M86512, M86519, M86521, M86522, M86529, M86531, M86532, M86539, M86541, M86542, M86549, M86551, M86552, M86559, M86561, M86562, M86569, M86571, M86572, M86579, M8658, M8659, M8660, M86611, M86612, M86619, M86621, M86622, M86629, M86631, M86632, M86639, M86641, M86642, M86649, M86651, M86652, M86659, M86661, M86662, M86669, M86671, M86672, M86679, M8668, M8669, M868X0, M868X1, M868X2, M868X3, M868X4, M868X5, M868X6, M868X7, M868X8, M868X9, M869 |  |
| Pyogenic Arthritis | M0000, M00011, M00012, M00019, M00021, M00022, M00029, M00031, M00032, M00039, M00041, M00042, M00049, M00051, M00052, M00059, M00061, M00062, M00069, M00071, M00072, M00079, M0008, M0009, M00111, M00112, M00119, M00121, M00122, M00129, M00131, M00132, M00139, M00141, M00142, M00149, M00151, M00152, M00159, M00161, M00162, M00169, M00171, M00172, M00179, M0018, M0019, M0020, M00211, M00212, M00219, M00221, M00222, M00229, M00231, M00232, M00239, M00241, M00242, M00249, M00251, M00252, M00259, M00261, M00262, M00269, M00271, M00272, M00279, M0028, M0029, M0080, M00811, M00812, M00819, M00821, M00822, M00829, M00831, M00832, M00839, M00841, M00842, M00849, M00851, M00852, M00859, M00861, M00862, M00869, M00871, M00872, M00879, M0088, M0089, M009 |  |
| Acute kidney injury | N170, N171, N172, N178, N179 |  |
| Infective Pericarditis | I301 |  |
| Altered mental status | R4182 |  |
| Obstruction of bile duct | K831 |  |
| Acute pancreatitis | K8500, K8501, K8502, K8510, K8511, K8512, K8580, K8581, K8582, K8590, K8591, K8592 |  |
| Vein Thrombosis | I82210, I82211, I82220, I82221, I82290, I82291, I823, I82401, I82402, I82403, I82409, I82411, I82412, I82413, I82419, I82421, I82422, I82423, I824, I82409, I82411, I82412, I82413, I82419, I82421, I82422, I82423, I82429, I82431, I82432, I82433, I82439, I82441, I82442, I82443, I82449, I82451, I82452, I82453, I82459, I82461, I82462, I82463, I82469, I82491, I82492, I82493, I82499, I824Y1, I824Y2, I824Y3, I824Y9, I824Z1, I824Z2, I824Z3, I824Z9, I82501, I82502, I82503, I82509, I82511, I82512, I82513, I82519, I82521, I82522, I82523, I82529, I82531, I82532, I82533, I82539, I82541, I82542, I82543, I82549, I82551, I82552, I82553, I82559, I82561, I82562, I82563, I82569, I82591, I82592, I82593, I82599, I825Y1, I825Y2, I825Y3, I825Y9, I825Z1, I825Z2, I825Z3, I825Z9, I82601, I82602, I82603, I82609, I82611, I82612, I82613, I82619, I82621, I82622, I82623, I82629, I82701, I82702, I82703, I82709, I82711, I82712, I82713, I82719, I82721, I82722, I82723, I82729, I82A11, I82A12, I82A13, I82A19, I82A21, I82A22, I82A23, I82A29, I82B11, I82B12, I82B13, I82B19, I82B21, I82B22, I82B23, I82B29, I82C11, I82C12, I82C13, I82C19, I82C21, I82C22, I82C23, I82C29, I82811, I82812, I82813, I82819, I82890, I82891, I82890, I82891 |  |
| Phlebitis and thrombophlebitis | I80 |  |
| Acute and subacute Endocarditis | I339, I330 |  |
| Septic arterial embolism | I76 |  |
| Pulmonary embolism | I2601, I2602, I2609, I2690, I2692, I2693, I2694, I2699 |  |
| **Procedure** | **ICD-10-PCS** |  |
| **Exclusion Criteria** |  |  |
| Immunosuppressed | 02YA0Z0, 02YA0Z2, 0BYC0Z0, 0BYC0Z2, 0BYD0Z0, 0BYD0Z2, 0BYF0Z0, 0BYF0Z2, 0BYG0Z0, 0BYG0Z2, 0BYH0Z0, 0BYH0Z2, 0BYJ0Z0, 0BYJ0Z2, 0BYK0Z0, 0BYK0Z2, 0BYL0Z0, 0BYL0Z2, 0BYM0Z0, 0BYM0Z2, 0DY50Z0, 0DY50Z2, 0DY60Z0, 0DY60Z2, 0DY80Z0, 0DY80Z2, 0DYE0Z0, 0DYE0Z2, 0FY00Z0, 0FY00Z2, 0FYG0Z0, 0FYG0Z2, 0TY00Z0, 0TY00Z2, 0TY10Z0, 0TY10Z2, 0WY20Z0, 0XYJ0Z0, 0XYK0Z0, 30230AZ, 30230G0, 30230G1, 30230G2, 30230G3, 30230G4, 30230U2, 30230U3, 30230U4, 30230X0, 30230X1, 30230X2, 30230X3, 30230X4, 30230Y0, 30230Y1, 30230Y2, 30230Y3, 30230Y4, 30233AZ, 30233G0, 30233G1, 30233G2, 30233G3, 30233G4, 30233U2, 30233U3, 30233U4, 30233X0, 30233X1, 30233X2, 30233X3, 30233X4, 30233Y0, 30233Y1, 30233Y2, 30233Y3, 30233Y4, 30240AZ, 30240G0, 30240G1, 30240G2, 30240G3, 30240G4, 30240U2, 30240U3, 30240U4, 30240X0, 30240X1, 30240X2, 30240X3, 30240X4, 30240Y0, 30240Y1, 30240Y2, 30240Y3, 30240Y4, 30243AZ, 30243G0, 30243G1, 30243G2, 30243G3, 30243G4, 30243U2, 30243U3, 30243U4, 30243X0, 30243X1, 30243X2, 30243X3, 30243X4, 30243Y0, 30243Y1, 30243Y2, 30243Y3, 30243Y4, 3E03005, 3E0300P, 3E030U1, 3E030WL, 3E03305, 3E0330P, 3E033U1, 3E033WL, 3E04005, 3E0400P, 3E040WL, 3E04305, 3E0430P, 3E043WL, 3E0A305, 3E0J3U1, 3E0J7U1, 3E0J8U1, XW01318, XW01348, XW03336, XW03351, XW03358, XW03368, XW03378, XW03387, XW03388, XW033B3, XW033C6, XW033D6, XW033H7, XW033J7, XW033K7, XW033M7, XW033N7, XW033S5, XW04336, XW04351, XW04358, XW04368, XW04378, XW04387, XW04388, XW043B3, XW043C6, XW043D6, XW043H7, XW043J7, XW043K7, XW043M7, XW043N7, XW043S5, XW133B8, XW133C8, XW143B8, XW143C8, XW23346, XW23376, XW24346, XW24376, |  |
| Mechanical Ventilation | F024GCZ, F025GCZ, F026GCZ, F027GCZ, F028GCZ, F029GCZ, F02BGCZ, F02CGCZ |  |

**Table S2.** Demographic and clinical characteristics of the overall hospitalization cohort

|  | **Overall** |
| --- | --- |
| **Hospitalizations, count** |  |
| Observed (Unweighted) | 3,967 |
| National (Weighted) | 19,835 |
| **Race, %** |  |
| White | 61.4 |
| Black | 23.9 |
| Hispanic | 9.5 |
| Other | 5.1 |
| **Weekend Admission, %** | 22.2 |
| **Hospital Location/Teaching Status, %** |  |
| Rural | 4.7 |
| Urban Nonteaching | 15.2 |
| Urban Teaching | 80.1 |
| **Hospital Region, %** |  |
| Northeast | 16.4 |
| Midwest | 22.4 |
| South | 44.2 |
| West | 17.1 |
| **Age, median [IQR]** | 56 [39-67] |
| 18-64, % | 68.8 |
| 65+, % | 31.2 |
| **Biological Sex, %** |  |
| Male | 51.8 |
| Female | 48.2 |
| **Primary Payer, %** |  |
| Medicare | 45.0 |
| Medicaid | 26.5 |
| Private | 21.4 |
| Other | 7.1 |
| **Comorbid Conditions, %** |  |
| Alcohol dependence | 6.1 |
| Asplenia | * |
| Burns | 0.6 |
| History of CAD | 21.4 |
| History of malignant neoplasm | 7.1 |
| Cerebral palsy | 1.0 |
| Chronic corticosteroid use | 2.6 |
| Cirrhosis of the liver | 8.1 |
| CKD | 16.2 |
|  | **Overall** |
| Congestive Heart Failure | 20.3 |
| Crohn's | 2.1 |
| Cystic fibrosis | 0.3 |
| Dementia | 3.8 |
| Depression | 16.8 |
| Diabetes Mellitus |  |
| Uncomplicated | 9.3 |
| Complicated | 23.1 |
| Dyslipidemia | 25.3 |
| Gallstones | 1.7 |
| GERD | 17.0 |
| Hemiplegia | 2.2 |
| Hemodialysis | 0.0 |
| HIV/AIDS | 0.0 |
| Hypertension | 48.3 |
| IBS | 0.9 |
| Ischemic heart disease | 19.0 |
| Left Ventricular systolic Dysfunction | * |
| Malnutrition | 19.2 |
| Mechanical Ventilation/tracheostomy | 5.5 |
| History of MI | 4.9 |
| Nephrolithiasis | 1.1 |
| Obesity | 24.1 |
| Paraplegia | 2.7 |
| Pulmonary hypertension | 4.8 |
| PVD | 8.5 |
| Quadriplegia | 2.0 |
| Sickle cell | 5.8 |
| Spinal cord injury | * |
| Stroke/TIA | 5.5 |
| History of Transplant | 0.0 |
| Ulcerative colitis | 0.7 |
| **Complications, %** |  |
| Sepsis | 61.1 |
| Septic shock | 21.1 |
| Severe Sepsis | 7.3 |
| Resistance to unspecified antimicrobial drugs | * |
| Mild cognitive impairment | * |
| Polyneuropathy | 1.7 |
| Acute myocardial infarction | 6.8 |
|  | **Overall** |
| Cerebral infarction, embolic and thrombotic | 3.4 |
| Cardiogenic shock | 5.3 |
| Acute respiratory distress syndrome | 4.7 |
| Disseminated intravascular coagulation | 1.5 |
| Infective myocarditis | * |
| Pneumonia | 24.1 |
| Urinary tract infection | 17.7 |
| Osteomyelitis | 7.2 |
| Pyogenic Arthritis | 3.1 |
| Acute kidney injury | 44.2 |
| Infective Pericarditis | * |
| Altered mental status | 0.4 |
| Obstruction of bile duct | 0.6 |
| Acute pancreatitis | 2.7 |
| Vein thrombosis | 14.7 |
| Phlebitis and thrombophlebitis | 2.3 |
| Acute and subacute Endocarditis | 5.6 |
| Septic arterial embolism | 1.5 |
| Pulmonary embolism | 5.2 |

*Note*. Data presented as count, percent, or median [IQR]. An * indicates that the data could not be presented per the NIS Data Use Agreement (the observed [unweighted] hospitalization count was < 11).

**Table S3.** Demographic and clinical characteristics stratified by race/ethnicity

|  | **White** | **Black** | **Hispanic** | **Other** | **p** |
| --- | --- | --- | --- | --- | --- |
| **Hospitalizations, count** |  |  |  |  |  |
| Observed (Unweighted) | 2,361 | 918 | 367 | 197 | - |
| National (Weighted) | 11,805 | 4,590 | 1,835 | 985 | - |
| **Weekend Admission, %** | 22.4 | 22.1 | 22.1 | 20.3 | 0.918 |
| **Hospital Location/Teaching Status, %** |  |  |  |  |  |
| Rural | 6.3 | 2.3 | * | * | * |
| Urban Nonteaching | 16.3 | 12.6 | 16.1 | 16.2 |  |
| Urban Teaching | 77.4 | 85.1 | 82.8 | 82.2 |  |
| **Hospital Region, %** |  |  |  |  |  |
| Northeast | 18.0 | 14.3 | 12.0 | 17.3 | <.001 |
| Midwest | 24.3 | 23.6 | 7.1 | 12.7 |  |
| South | 41.3 | 54.1 | 47.1 | 33.0 |  |
| West | 16.4 | 8.0 | 33.8 | 37.1 |  |
| **Age, median [IQR]** | 58 [44-69] | 49 [34-62] | 56 [38-67] | 56 [38-69] | <.001 |
| 18-64, % | 64.7 | 79.2 | 70.0 | 62.9 | <.001 |
| 65+, % | 35.3 | 20.8 | 30.0 | 37.1 |  |
| **Biological Sex, %** |  |  |  |  |  |
| Male | 53.2 | 44.8 | 60.8 | 53.8 | <.001 |
| Female | 46.8 | 55.2 | 39.2 | 46.2 |  |
| **Primary Payer, %** |  |  |  |  |  |
| Medicare | 48.6 | 41.9 | 37.1 | 37.1 | <.001 |
| Medicaid | 21.9 | 35.4 | 33.0 | 29.9 |  |
| Private | 22.7 | 17.0 | 19.6 | 24.9 |  |
| Other | 6.9 | 5.7 | 10.4 | 8.0 |  |
| **Comorbid Conditions, %** |  |  |  |  |  |
| Alcohol dependence | 7.0 | 4.0 | 4.9 | 6.6 | 0.011 |
| Asplenia | * | * | * | * | * |
| Burns | * | * | * | * | * |
|  | **White** | **Black** | **Hispanic** | **Other** | **p** |
| History of CAD | 23.5 | 18.3 | 17.2 | 19.3 | 0.001 |
| History of malignant neoplasm | 8.4 | 5.4 | 5.4 | * | * |
| Cerebral palsy | 0.8 | 1.7 | * | * | * |
| Chronic corticosteroid use | 3.1 | 2.3 | * | * | * |
| Cirrhosis of the liver | 9.4 | 4.4 | 9.0 | 7.6 | <.001 |
| CKD | 15.7 | 17.2 | 16.1 | 15.2 | 0.744 |
| Congestive Heart Failure | 21.1 | 19.0 | 16.6 | 21.8 | 0.161 |
| Crohn's | 2.5 | 1.9 | * | * | * |
| Cystic fibrosis | 0.5 | * | * | * | * |
| Dementia | 3.1 | 4.9 | 4.4 | 6.1 | 0.021 |
| Depression | 18.6 | 13.8 | 12.3 | 12.2 | <.001 |
| Diabetes Mellitus |  |  |  |  |  |
| Uncomplicated | 9.3 | 8.6 | 11.7 | 7.1 | 0.248 |
| Complicated | 22.3 | 21.8 | 28.3 | 26.9 | 0.031 |
| Dyslipidemia | 26.9 | 20.8 | 26.7 | 24.9 | 0.004 |
| Gallstones | 1.9 | 1.2 | * | * | * |
| GERD | 19.3 | 13.9 | 11.4 | 10.7 | <.001 |
| Hemiplegia | 1.8 | 2.3 | 3.8 | * | * |
| Hemodialysis | 0.0 | 0.0 | 0.0 | 0.0 | - |
| HIV/AIDS | 0.0 | 0.0 | 0.0 | 0.0 | - |
| Hypertension | 47.8 | 48.3 | 50.7 | 51.3 | 0.628 |
| IBS | 1.2 | * | * | * | * |
| Ischemic heart disease | 21.5 | 15.1 | 14.7 | 15.2 | <.001 |
| Left Ventricular systolic Dysfunction | * | * | * | * | * |
| Malnutrition | 19.8 | 17.5 | 15.8 | 18.8 | 0.191 |
| Mechanical Ventilation/tracheostomy | 4.6 | 6.0 | 6.8 | 11.2 | <.001 |
| History of MI | 5.7 | 4.1 | * | * | * |
| Nephrolithiasis | 1.2 | * | * | * | * |
| Obesity | 24.4 | 22.8 | 28.1 | 19.8 | 0.108 |
|  | **White** | **Black** | **Hispanic** | **Other** | **p** |
| Paraplegia | 2.6 | 3.5 | * | * | * |
| Pulmonary hypertension | 5.3 | 3.9 | 4.9 | * | * |
| PVD | 9.2 | 6.3 | 9.8 | 7.1 | 0.037 |
| Quadriplegia | 1.7 | 2.0 | 4.4 | * | * |
| Sickle cell | * | 23.3 | * | * | * |
| Spinal cord injury | * | * | * | * | * |
| Stroke/TIA | 4.7 | 9.0 | * | * | * |
| History of Transplant | 0.0 | 0.0 | 0.0 | 0.0 | - |
| Ulcerative colitis | 0.9 | * | * | * | * |
| **Complications, %** |  |  |  |  |  |
| Sepsis | 60.0 | 57.7 | 70.6 | 69.5 | <.001 |
| Septic shock | 20.8 | 15.5 | 32.2 | 28.4 | <.001 |
| Severe Sepsis | 7.4 | 7.8 | 6.8 | * | * |
| Resistance to unspecified antimicrobial drugs | * | * | * | * | * |
| Mild cognitive impairment | * | * | * | * | * |
| Polyneuropathy | 1.7 | * | 3.2 | * | * |
| Acute myocardial infarction | 7.1 | 5.3 | 7.6 | 10.2 | 0.066 |
| Cerebral infarction, embolic and thrombotic | 2.8 | 4.0 | 4.1 | * | * |
| Cardiogenic shock | 5.5 | 5.0 | 3.8 | 6.1 | 0.515 |
| Acute respiratory distress syndrome | 3.8 | 2.3 | 13.9 | 10.7 | <.001 |
| Disseminated intravascular coagulation | 1.3 | 1.3 | * | * | * |
| Infective myocarditis | * | * | * | * | * |
| Pneumonia | 23.2 | 20.7 | 34.9 | 33.5 | <.001 |
| Urinary tract infection | 17.2 | 18.2 | 19.9 | 18.3 | 0.588 |
| Osteomyelitis | 7.5 | 6.1 | 6.5 | 7.6 | 0.500 |
| Pyogenic Arthritis | 3.7 | 1.4 | * | * | * |
| Acute kidney injury | 43.7 | 42.5 | 50.7 | 46.7 | 0.054 |
| Infective Pericarditis | * | * | * | * | * |
| Altered mental status | * | * | * | * | * |
|  | **White** | **Black** | **Hispanic** | **Other** | **p** |
| Obstruction of bile duct | 0.6 | * | * | * | * |
| Acute pancreatitis | 3.0 | 1.7 | * | * | * |
| Vein thrombosis | 14.7 | 13.9 | 19.6 | 12.7 | 0.049 |
| Phlebitis and thrombophlebitis | 2.8 | 1.6 | * | * | * |
| Acute and subacute Endocarditis | 6.7 | 2.7 | 5.2 | 5.6 | <.001 |
| Septic arterial embolism | 1.8 | * | 3.0 | * | * |
| Pulmonary embolism | 5.9 | 4.5 | 4.4 | * | * |

*Note*. Data presented as count, percent, or median [IQR]. An * indicates that the data could not be presented per the NIS Data Use Agreement (the observed [unweighted] hospitalization count was < 11).

**Table S4.** Demographic and clinical characteristics stratified by location-teaching status

|  | **Rural** | **Urban**  **Non-Teaching** | **Urban Teaching** | **p** |
| --- | --- | --- | --- | --- |
| **Hospitalizations, count** |  |  |  |  |
| Observed (Unweighted) | 187 | 604 | 3,176 | - |
| National (Weighted) | 935 | 3,020 | 15,880 | - |
| **Race, %** |  |  |  |  |
| White | 84.2 | 65.0 | 59.4 | * |
| Black | 11.9 | 19.6 | 25.4 |  |
| Hispanic | * | 10.0 | 9.9 |  |
| Other | * | 5.4 | 5.3 |  |
| **Weekend Admission, %** | 25.1 | 21.9 | 22.0 | 0.579 |
| **Hospital Region, %** |  |  |  |  |
| Northeast | 9.6 | 9.1 | 18.2 | <.001 |
| Midwest | 30.5 | 13.7 | 23.6 |  |
| South | 50.3 | 54.1 | 41.9 |  |
| West | 9.6 | 23.0 | 16.4 |  |
| **Age, median [IQR]** | 60 [46-73] | 47 [42-69] | 55 [38-66] | <.001 |
| 18-64, % | 56.1 | 64.2 | 70.4 | <.001 |
| 65+, % | 43.9 | 35.8 | 29.6 |  |
| **Biological Sex, %** |  |  |  |  |
| Male | 52.9 | 48.7 | 52.3 | 0.228 |
| Female | 47.1 | 51.3 | 47.7 |  |
| **Primary Payer, %** |  |  |  |  |
| Medicare | 55.1 | 46.9 | 44.1 | * |
| Medicaid | 20.3 | 24.0 | 27.4 |  |
| Private | 20.3 | 22.0 | 21.3 |  |
| Other | * | 7.1 | 7.2 |  |
| **Comorbid Conditions, %** |  |  |  |  |
| Alcohol dependence | * | 6.8 | 6.0 | * |
|  | **Rural** | **Urban**  **Non-Teaching** | **Urban Teaching** | **p** |
| Asplenia | * | * | * | * |
| Burns | * | * | 0.7 | * |
| History of CAD | 21.4 | 21.2 | 21.4 | 0.993 |
| History of malignant neoplasm | 8.6 | 6.3 | 7.2 | 0.540 |
| Cerebral palsy | * | * | 0.9 | * |
| Chronic corticosteroid use | * | 1.8 | 2.9 | * |
| Cirrhosis of the liver | 8.0 | 7.5 | 8.3 | 0.783 |
| CKD | 17.6 | 17.5 | 15.8 | 0.474 |
| Congestive Heart Failure | 26.2 | 20.4 | 19.9 | 0.115 |
| Crohn's | * | * | 2.2 | * |
| Cystic fibrosis | * | * | 0.4 | * |
| Dementia | * | 3.6 | 3.7 | * |
| Depression | 20.9 | 15.9 | 16.7 | 0.248 |
| Diabetes Mellitus |  |  |  |  |
| Uncomplicated | 11.8 | 9.4 | 9.1 | 0.450 |
| Complicated | 27.3 | 24.3 | 22.6 | 0.221 |
| Dyslipidemia | 25.7 | 26.7 | 25.0 | 0.698 |
| Gallstones | * | * | 1.8 | * |
| GERD | 221.9 | 17.2 | 16.7 | 0.163 |
| Hemiplegia | * | 2.5 | 2.2 | 0.905 |
| Hemodialysis | 0.0 | 0.0 | 0.0 | - |
| HIV/AIDS | 0.0 | 0.0 | 0.0 | - |
| Hypertension | 53.5 | 51.8 | 47.3 | 0.033 |
| IBS | * | * | 0.9 | * |
| Ischemic heart disease | 20.9 | 19.0 | 18.8 | 0.793 |
| Left Ventricular systolic Dysfunction | * | * | * | * |
| Malnutrition | 24.6 | 18.5 | 19.0 | 0.147 |
| Mechanical Ventilation/tracheostomy | * | 6.0 | 5.5 | 0.692 |
|  | **Rural** | **Urban**  **Non-Teaching** | **Urban Teaching** | **p** |
| History of MI | * | 4.0 | 5.1 | 0.566 |
| Nephrolithiasis | * | 1.8 | 0.9 | * |
| Obesity | 22.5 | 23.2 | 24.4 | 0.684 |
| Paraplegia | * | 2.0 | 2.9 | * |
| Pulmonary hypertension | * | 5.0 | 4.8 | * |
| PVD | 9.1 | 7.8 | 8.6 | 0.790 |
| Quadriplegia | * | 3.3 | 1.8 | * |
| Sickle cell | * | 4.3 | 6.3 | * |
| Spinal cord injury | * | * | * | * |
| Stroke/TIA | * | 7.0 | 5.4 | * |
| History of Transplant | 0.0 | 0.0 | 0.0 | - |
| Ulcerative colitis | * | * | 0.7 | * |
| **Complications, %** |  |  |  |  |
| Sepsis | 55.1 | 64.4 | 60.8 | 0.073 |
| Septic shock | 16.0 | 21.0 | 21.4 | 0.261 |
| Severe Sepsis | 6.4 | 10.1 | 6.9 | 0.018 |
| Resistance to unspecified antimicrobial drugs | * | * | * | * |
| Mild cognitive impairment | * | * | * | * |
| Polyneuropathy | * | * | 1.9 | * |
| Acute myocardial infarction | * | 6.6 | 7.0 | * |
| Cerebral infarction, embolic and thrombotic | * | 4.0 | 3.2 | * |
| Cardiogenic shock | * | 3.0 | 6.0 | * |
| Acute respiratory distress syndrome | * | 4.0 | 4.8 | * |
| Disseminated intravascular coagulation | * | * | 1.5 | * |
| Infective myocarditis | * | * | * | * |
| Pneumonia | 23.0 | 27.6 | 23.6 | 0.090 |
| Urinary tract infection | 16.0 | 19.2 | 17.6 | 0.506 |
| Osteomyelitis | 7.0 | 7.1 | 7.2 | 0.989 |
|  | **Rural** | **Urban**  **Non-Teaching** | **Urban Teaching** | **p** |
| Pyogenic Arthritis | * | 3.0 | 3.1 | * |
| Acute kidney injury | 37.4 | 42.2 | 45.0 | 0.087 |
| Infective Pericarditis | * | * | * | * |
| Altered mental status | * | * | 0.5 | * |
| Obstruction of bile duct | * | * | 0.7 | * |
| Acute pancreatitis | * | 3.3 | 2.6 | * |
| Vein thrombosis | 8.0 | 12.6 | 15.5 | 0.005 |
| Phlebitis and thrombophlebitis | * | 2.5 | 2.3 | * |
| Acute and subacute Endocarditis | 6.4 | 4.6 | 5.7 | 0.479 |
| Septic arterial embolism | * | * | 1.6 | * |
| Pulmonary embolism | * | 4.8 | 5.4 | * |

*Note*. Data presented as count, percent, or median [IQR]. An * indicates that the data could not be presented per the NIS Data Use Agreement (the observed [unweighted] hospitalization count was < 11).

**Table S5.** Demographic and clinical characteristics stratified by geographic region

|  | **Northeast** | **Midwest** | **South** | **West** | **p** |
| --- | --- | --- | --- | --- | --- |
| **Hospitalizations, count** |  |  |  |  |  |
| Observed (Unweighted) | 650 | 888 | 1,752 | 677 | - |
| National (Weighted) | 3,250 | 4,440 | 8,760 | 3,385 | - |
| **Race, %** |  |  |  |  |  |
| White | 67.1 | 68.1 | 57.0 | 58.9 | <.001 |
| Black | 20.6 | 25.8 | 29.1 | 11.1 |  |
| Hispanic | 6.9 | 3.1 | 10.1 | 18.9 |  |
| Other | 5.4 | 3.0 | 3.8 | 11.1 |  |
| **Weekend Admission, %** | 21.2 | 22.3 | 22.5 | 22.0 | 0.926 |
| **Hospital Location/Teaching Status, %** |  |  |  |  |  |
| Rural | 2.8 | 6.4 | 5.4 | 2.7 | <.001 |
| Urban Nonteaching | 8.5 | 9.3 | 18.7 | 20.5 |  |
| Urban Teaching | 88.8 | 84.2 | 76.0 | 76.8 |  |
| **Age, median [IQR]** | 58 [39-68] | 56 [41-68] | 55 [39-66] | 55 [39-67] | 0.265 |
| 18-64, % | 65.7 | 67.2 | 70.8 | 68.7 | 0.056 |
| 65+, % | 34.3 | 32.8 | 29.2 | 31.3 |  |
| **Biological Sex, %** |  |  |  |  |  |
| Male | 49.5 | 48.5 | 53.8 | 53.2 | 0.036 |
| Female | 50.5 | 51.5 | 46.2 | 46.8 |  |
| **Primary Payer, %** |  |  |  |  |  |
| Medicare | 45.9 | 49.0 | 44.5 | 40.2 | <.001 |
| Medicaid | 26.0 | 26.2 | 23.9 | 34.1 |  |
| Private | 23.1 | 19.9 | 22.0 | 20.1 |  |
| Other | 4.9 | 4.8 | 9.5 | 5.6 |  |
| **Comorbid Conditions, %** |  |  |  |  |  |
| Alcohol dependence | 5.5 | 6.9 | 5.0 | 8.6 | 0.005 |
| Asplenia | * | * | * | * | * |
| Burns | * | * | 1.0 | * | * |
|  | **Northeast** | **Midwest** | **South** | **West** | **p** |
| History of CAD | 21.2 | 26.9 | 20.8 | 15.8 | <.001 |
| History of malignant neoplasm | 6.9 | 9.9 | 6.6 | 5.0 | 0.003 |
| Cerebral palsy | * | 1.2 | 0.8 | * | * |
| Chronic corticosteroid use | 2.6 | 3.9 | 2.0 | 2.7 | 0.033 |
| Cirrhosis of the liver | 5.7 | 8.9 | 8.2 | 9.3 | 0.063 |
| CKD | 14.5 | 19.1 | 16.6 | 12.7 | 0.004 |
| Congestive Heart Failure | 18.2 | 22.1 | 21.0 | 18.2 | 0.120 |
| Crohn's | 1.8 | 2.5 | 2.2 | 1.8 | 0.724 |
| Cystic fibrosis | * | * | * | * | * |
| Dementia | 3.5 | 3.8 | 4.3 | 2.7 | 0.312 |
| Depression | 16.5 | 22.6 | 14.4 | 15.5 | <.001 |
| Diabetes Mellitus |  |  |  |  |  |
| Uncomplicated | 10.3 | 10.1 | 9.1 | 7.5 | 0.228 |
| Complicated | 21.8 | 25.2 | 22.9 | 21.9 | 0.320 |
| Dyslipidemia | 27.2 | 33.7 | 22.5 | 19.6 | <.001 |
| Gallstones | * | 2.1 | 1.3 | 2.4 | * |
| GERD | 16.3 | 22.3 | 15.4 | 14.9 | <.001 |
| Hemiplegia | 1.8 | 2.0 | 2.3 | 2.7 | 0.725 |
| Hemodialysis | 0.0 | 0.0 | 0.0 | 0.0 | - |
| HIV/AIDS | 0.0 | 0.0 | 0.0 | 0.0 | - |
| Hypertension | 44.3 | 51.9 | 47.8 | 43.4 | 0.001 |
| IBS | * | 1.2 | 0.7 | * | * |
| Ischemic heart disease | 18.8 | 24.2 | 18.9 | 12.4 | <.001 |
| Left Ventricular systolic Dysfunction | * | * | * | * | * |
| Malnutrition | 15.7 | 26.5 | 16.3 | 20.8 | <.001 |
| Mechanical Ventilation/tracheostomy | 7.1 | 5.1 | 4.5 | 7.2 | 0.019 |
| History of MI | 4.2 | 8.6 | 3.7 | 4.3 | <.001 |
| Nephrolithiasis | * | 1.4 | 1.0 | * | * |
| Obesity | 22.8 | 29.6 | 22.1 | 23.5 | <.001 |
|  | **Northeast** | **Midwest** | **South** | **West** | **p** |
| Paraplegia | * | 2.9 | 2.9 | 3.7 | * |
| Pulmonary hypertension | 4.6 | 4.8 | 4.5 | 5.6 | 0.718 |
| PVD | 7.8 | 9.2 | 8.1 | 9.0 | 0.656 |
| Quadriplegia | 2.5 | 2.1 | 2.0 | 1.6 | 0.748 |
| Sickle cell | 6.0 | 5.7 | 6.6 | 3.5 | 0.068 |
| Spinal cord injury | * | * | * | * | * |
| Stroke/TIA | 3.7 | 6.5 | 6.1 | 4.6 | 0.048 |
| History of Transplant | 0.0 | 0.0 | 0.0 | 0.0 | - |
| Ulcerative colitis | * | * | 0.7 | * | * |
| **Complications, %** |  |  |  |  |  |
| Sepsis | 57.8 | 57.7 | 62.2 | 65.7 | 0.004 |
| Septic shock | 21.4 | 17.9 | 21.4 | 24.4 | 0.028 |
| Severe Sepsis | 7.5 | 7.1 | 7.6 | 6.6 | 0.851 |
| Resistance to unspecified antimicrobial drugs | * | * | * | * | * |
| Mild cognitive impairment | * | * | * | * | * |
| Polyneuropathy | 1.7 | 1.7 | 1.5 | 2.2 | 0.650 |
| Acute myocardial infarction | 7.2 | 5.5 | 7.1 | 7.4 | 0.343 |
| Cerebral infarction, embolic and thrombotic | 2.6 | 3.2 | 3.9 | 3.1 | 0.402 |
| Cardiogenic shock | 5.8 | 5.3 | 5.4 | 4.6 | 0.772 |
| Acute respiratory distress syndrome | 5.1 | 5.1 | 3.9 | 5.9 | 0.196 |
| Disseminated intravascular coagulation | * | * | 1.8 | 1.8 | * |
| Infective myocarditis | * | * | * | * | * |
| Pneumonia | 22.0 | 23.1 | 24.5 | 26.7 | 0.209 |
| Urinary tract infection | 15.4 | 16.4 | 19.5 | 17.1 | 0.055 |
| Osteomyelitis | 6.0 | 7.2 | 7.8 | 6.8 | 0.502 |
| Pyogenic Arthritis | 2.8 | 2.9 | 3.2 | 3.2 | 0.934 |
| Acute kidney injury | 44.9 | 42.6 | 46.2 | 40.8 | 0.087 |
| Infective Pericarditis | * | * | * | * | * |
| Altered mental status | * | * | * | * | * |
|  | **Northeast** | **Midwest** | **South** | **West** | **p** |
| Obstruction of bile duct | * | * | 0.7 | * | * |
| Acute pancreatitis | 2.8 | 2.4 | 2.7 | 2.8 | 0.928 |
| Vein thrombosis | 15.7 | 14.1 | 14.0 | 16.4 | 0.410 |
| Phlebitis and thrombophlebitis | 2.8 | 2.4 | 2.0 | 2.7 | 0.643 |
| Acute and subacute Endocarditis | 5.1 | 4.2 | 6.2 | 6.2 | 0.120 |
| Septic arterial embolism | * | * | 1.7 | 2.1 | * |
| Pulmonary embolism | 4.5 | 5.3 | 5.3 | 5.6 | 0.800 |

*Note*. Data presented as count, percent, or median [IQR]. An * indicates that the data could not be presented per the NIS Data Use Agreement (the observed [unweighted] hospitalization count was < 11).
